# Variation in sensitivity of large benthic Foraminifera to the combined effects of ocean warming and local impacts

**DOI:** 10.1038/srep45227

**Published:** 2017-03-23

**Authors:** Martina Prazeres, T. Edward Roberts, John M. Pandolfi

**Affiliations:** 1 ARC Centre of Excellent for Coral Reef Studies and School of Biological Sciences, The University of Queensland, St. Lucia, QLD 4072, Australia; 2 ARC Centre of Excellent for Coral Reef Studies, James Cook University, Townsville, QLD 4811, Australia

## Abstract

Large benthic foraminifera (LBF) are crucial marine calcifiers in coral reefs, and sensitive to environmental changes. Yet, many species successfully colonise a wide range of habitats including highly fluctuating environments. We tested the combined effects of ocean warming, local impacts and different light levels on populations of the common LBF *Amphistegina lobifera* collected along a cross-shelf gradient of temperature and nutrients fluctuations. We analysed survivorship, bleaching frequency, chlorophyll a content and fecundity. Elevated temperature and nitrate significantly reduced survivorship and fecundity of *A. lobifera* across populations studied. This pattern was exacerbated when combined with below optimum light levels. Inshore populations showed a consistent resistance to increased temperature and nitrate levels, but all populations studied were significantly affected by light reduction. These findings demonstrated the capacity of some populations of LBF to acclimate to local conditions; nonetheless improvements in local water quality can ultimately ameliorate effects of climate change in local LBF populations.

Marine environments are projected to change at unprecedented rates due to global warming and local disturbances^1^,^2^. It remains unclear if reliable predictions of climate change impacts on marine populations and ecosystems can be made, due to insufficient understanding of the capacity for marine organisms to adapt to climate change and the interactions with local stressors^3^,^4^. Indeed, the unusual rate and extent of anthropogenic alterations of the environment may exceed the evolutionary capacity of populations to deal with environmental changes^5^.Coral reefs are among the marine ecosystems that are experiencing rapid degradation driven by both climate change and local impacts such as overfishing and land runoff^6^,^7^. Nevertheless, predicting the fate of reef organisms in light of these changes requires detailed understanding of their ability to acclimate or even adapt to rapid shifts in environmental conditions^8^.

Phenotypic plasticity is one mechanism that allows organisms to adjust their physiology and morphology according to local conditions^9^. There is ample evidence that populations of the same morpho-species when experiencing different environmental conditions often differ in phenotypic characteristics and genetic structure^10^. Phenotypic plasticity is advantageous as it enables a species to adjust to changes in local conditions and occupy a broad range of habitats^11^. This also provides the potential for organisms to respond rapidly and effectively to local environmental changes^12^.

Large benthic foraminifera (LBF) are a group of single-celled protists that build a calcium carbonate (CaCO_3_) test, producing mainly calcitic tests^13^, and harbor algae as symbionts, which provides them with energy for growth and calcification^14^. They are prolific marine calcifiers, and significant contributors to CaCO_3_ cycling in the ocean^15^. Their geographic distribution is largely limited to oligotrophic waters of tropical and warm temperate seas^14^,^16^. However, many LBF populations are able to live in marginal and highly fluctuating environments^17^. Due to their unicellular nature, LBFs respond fast to environmental changes^18^, and their small size and short life spans make them an ideal model system to study the response of holobiont (i.e., host and photosymbiont) reef organisms to changes in environmental conditions.

Among coral reef-dwelling LBFs, foraminifera from the genus *Amphistegina* are the most widely distributed and abundant worldwide^14^. They can live over a wide depth range (<1 m to ~100 m) using phototaxic behaviour^19^. Additionally, species of *Amphistegina* can display changes in thickness and shape of the test according to depth of occurrence, which highlights the phenotypic plasticity within the genus^20^. *Amphistegina* utilise a reticulopodial network to move across the substrate^21^. This allows cryptic behaviour to avoid damage from intense light in shallow water^14^, and to attain suitable light exposure^19^,^21^, with only ~1% of tropical sea-surface light intensity required for optimal growth in laboratory conditions^22^,^23^.

*Amphistegina* are sensitive to thermal and photic stress, and can be particularly sensitive to minimal exposure to ultraviolet B radiation, leading to symbiont loss (i.e., bleaching)^24^. Additionally, long-term exposure to temperatures above 28 °C inducing bleaching in populations of *A. gibbosa*^24^ and *A. radiata*^25^. Recent studies demonstrate that while conspecific populations of the LBF *A. lobifera* living in different reef locations respond differently to elevated temperatures^26^,^27^, they showed consistent responses to changing light levels^28^. These studies suggest that LBFs have developed mechanisms to quickly (i.e., within days) acclimate to changes in light that are independent of their local habitat^28^, but the capacity to acclimate to increases in temperature is likely to be linked to the life history of each individual population^26^.

*Amphistegina* are well adapted to live under low-nutrient conditions due to their symbiotic relationship with algal endosymbionts^29^, as symbionts provide the host-symbiont system with fixed carbon when dissolved inorganic nutrients are low in concentration. However, changes in nutrient loads have the potential to disrupt the finely balanced nutrient cycling pathways of the symbiotic relationship, making this species particularly sensitive to macroalgal overgrowth under elevated nutrients^30^. Previous field-based experiments showed that increased nutrient concentrations were associated with reduced growth rates in *A. radiata*^31^. However, the sensitivity of *A. lobifera* to increases in nitrate levels (up to 4.5 μM) can also be dependent on their local habitat^26^, and populations adapted to regular pulses of nutrients are able to tolerate mesotrophic conditions.

Here, we use the LBF morpho-species *A. lobifera* as a model organism to study the influence of local habitat in the responses of holobiont systems to the combined effects of ocean warming and local impacts. We compared populations that have previously showed differential sensitivity to the independent effects of elevated temperature or nitrate^26^. We used an experimental approach to analyse biological parameters of survivorship, bleaching frequency, chlorophyll *a* (Chl *a*) content and fecundity of *A. lobifera* individuals collected from different reef sites. Specimens were exposed to the combined effects of different conditions of temperature and nitrate in two independent full orthogonal design experiments using two different light levels. This approach allows us to significantly improve our understanding of how predicted changes in the local environment, caused by the combinations of local and global impacts, are likely to affect the life history and biology of *Amphistegina* populations and possibly other holobiont systems.

## Results

### Survivorship and bleaching frequency in *A. lobifera*

#### Experiment 1: control light levels

**Figure 1:**
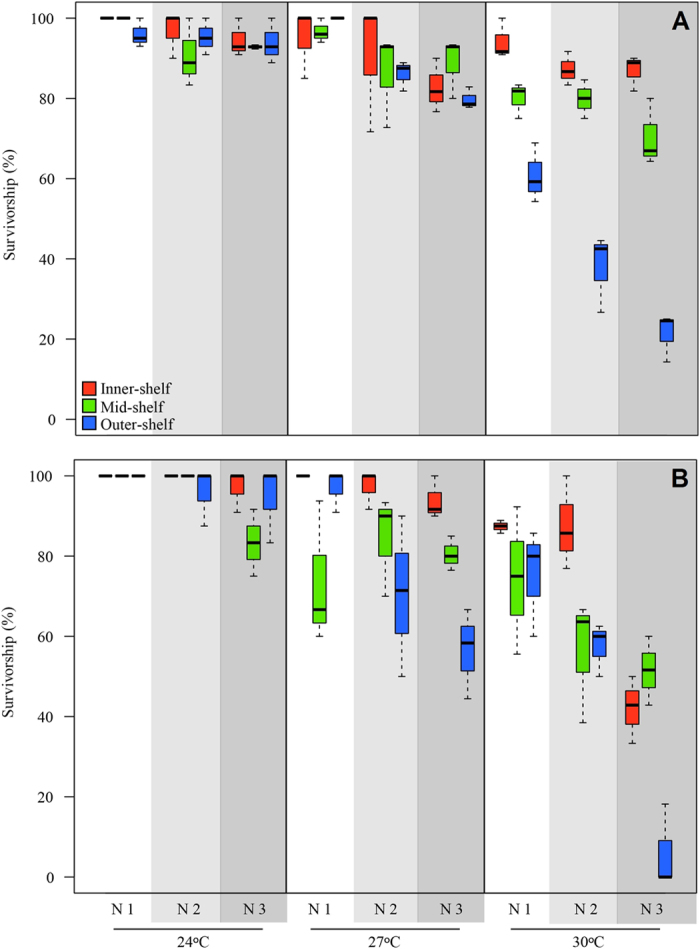
Survivorship (%) of populations collected from different reef sites, and exposed to the combined effects of elevated temperature and nitrate after 30 days. (**A**) Control light levels of 80 μE m^2−^ s^−^. (**B**) Low light levels of 30 μE m^2−^ s^−^. Different shades of grey represent different nitrate concentrations. N1, N2 and N3 represent 0.09, 1.1 and 3.2 μM NO_3_^−^, respectively.

The independent effect of temperature, nitrate and reef sites was not significant on survivorship of *A. lobifera*. However, the combined effect of reef site and temperature had a significant influence on survivorship (Table 1; Supplementary Table S2). The interaction of reef site*nitrate, temperature*nitrate and reef site* temperature*nitrate were not significant. Survivorship declined significantly only in the outer-shelf reef population exposed to high temperature (Fig. 1A), reaching an average of ~25% at the highest temperature and nitrate tested.

**Figure 2:**
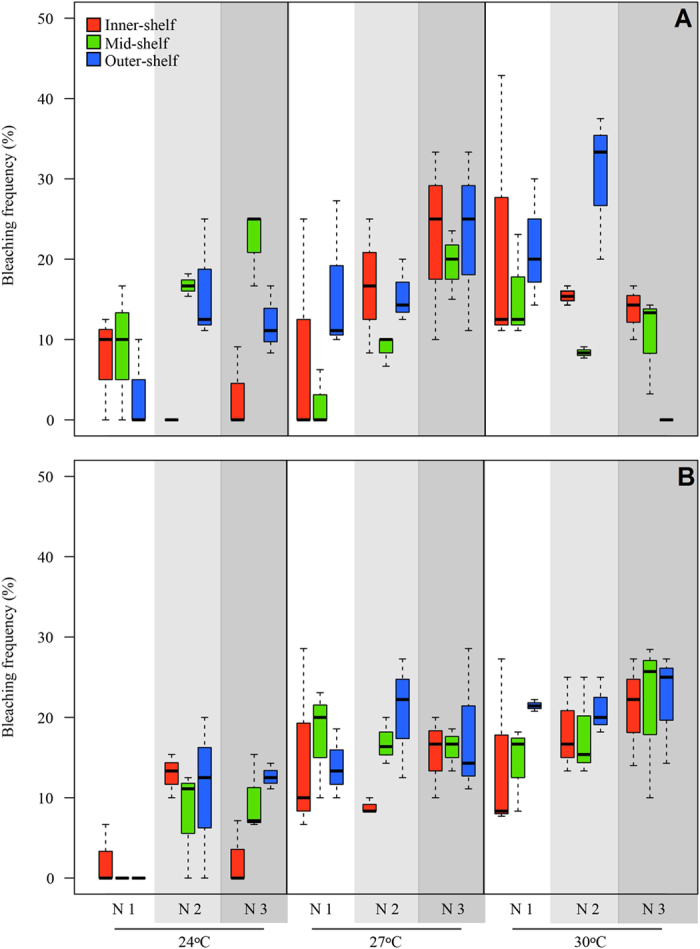
Bleaching frequency (%) of populations collected from different reef sites, and exposed to the combined effects of elevated temperature and nitrate after 30 days. (**A**) Control light levels of 80 μE m^2−^ s^−^. (**B**) Low light levels of 30 μE m^2−^ s^−^. Different shades of grey represent different nitrate concentrations. N1, N2 and N3 represent 0.09, 1.1 and 3.2 μM NO_3_^−^, respectively.

Bleaching frequency varied significantly between treatments and reef sites. The generalised linear mixed model showed that the individual effects of both temperature and reef site were significant (Table 2, Supplementary Table S3), while nitrate did not have a significant effect. The interaction of the three factors was significant (Table 2). The combined effect of temperature and nitrate had the strongest influence on bleaching responses in *A. lobifera* across reef sites (Fig. 2A). Bleaching significantly increased in the outer-shelf reef population with elevated temperature and nitrate, to above an average of ~20%, but was lower at inner- and mid-shelf reef populations, which showed bleaching frequency of ~15%.

**Figure 3:**
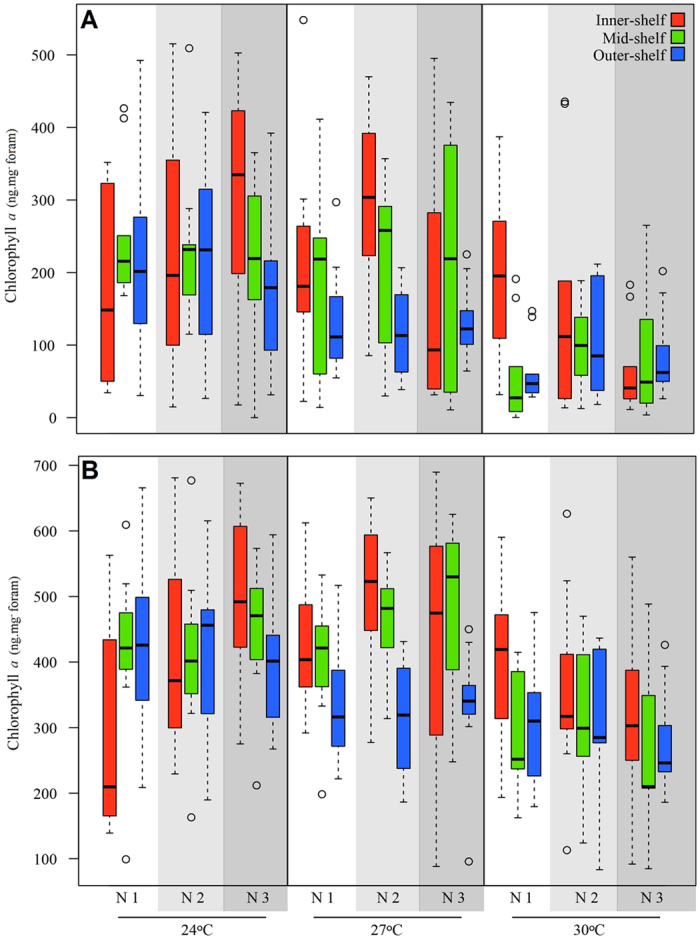
Chlorophyll *a* concentration of individuals of populations collected from different reef sites, and exposed to the combined effects of elevated temperature and nitrate after 30 days. (**A**) Control light levels of 80 μE m^2−^ s^−^. (**B**) Low light levels of 30 μE m^2−^ s^−^. Different shades of grey represent different nitrate concentrations. N1, N2 and N3 represent 0.09, 1.1 and 3.2 μM NO_3_^−^, respectively.

Chlorophyll *a* content varied significantly between temperature treatments and reef sites, but nitrate showed no significant interaction (Table 3, Supplementary Table S4). The interaction of reef site*nitrate*temperature was also not significant. Chlorophyll *a* concentration declined significantly at elevated temperatures across all reef sites (Fig. 3A), consistent with the bleaching frequency results. The highest average Chl *a* of 300 ng mg^−^ per foram was detected in the inner-shelf population exposed to ambient temperature and the highest nitrate level.

#### Experiment 2: reduced light levels

Under reduced light levels, survivorship of populations of *A. lobifera* collected from different reef sites was not significantly affected by the single effect of temperature or nitrate. The interactions between reef site*temperature and reef site*temperature*nitrate were significant (Table 4, Supplementary Table S5). Inner-shelf and mid-shelf populations were mildly affected, and even when exposed to the highest temperature and nitrate levels survivorship remained ~50% (Fig. 1B).

The outer-shelf population was severely affected by the interaction of temperature and nitrate, and survivorship dropped from 100% (ambient conditions of temperature and nitrate) to an average of ~5% when exposed to high temperature and nitrate levels (Fig. 1B).

Bleaching frequency was variable across treatments, and the single effect of temperature or nitrate was significant, as well as the interaction between these factors (Table 5, Supplementary Table S6). In contrast to populations that were exposed to control light levels, average bleaching frequency under reduced light remained below 25% across all treatments and reef sites. The highest average bleaching frequency (22%) was detected in the outer-shelf population exposed to the highest temperature and nitrate levels (Fig. 2B).

Chlorophyll *a* content of foraminifera showed no significant differences across reef sites and treatments (Table 6, Supplementary Table S7), and content in the individuals exposed to reduced light levels was, in general, higher than in those exposed to control light levels. Highest and lowest Chl *a* concentration were ~500 and 275 ng mg^−^ foram, respectively (Fig. 3B).

### Fecundity across reef sites and treatments

Fecundity varied across reef sites and treatments. However, reduced light had a clear negative effect on fecundity of *A. lobifera*. Under control light levels, a total of 55 individuals underwent asexual reproduction across different reef site populations and treatments, as opposed to only seven specimens under reduced light levels (Supplementary Table S1). Moreover, only inner-shelf individuals produced newborns and only under ambient conditions of temperature and nitrate (Supplementary Table S1).

**Figure 4:**
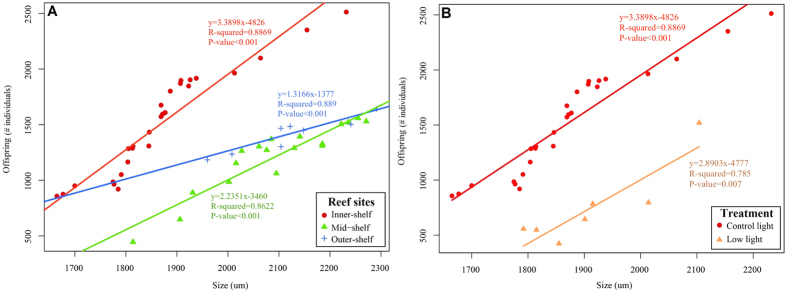
Fecundity of asexual reproduction as a function of adult test size in individuals collected from different reef sites, and exposed to the combined effects of elevated temperature and nitrate after 30 days. (**A**) Comparison between inner-, mid- and outer-shelf individuals across all temperature*nitrate treatments under control light levels. (**B**) Comparison between inner-shelf individuals exposed to control and low light levels, across all temperature*nitrate treatments.

Comparison between slopes showed significant differences between number of offspring and size of adults at reef sites exposed to control light conditions (Table 7). Inner-shelf specimens produced a higher number of individuals through asexual reproduction at any given adult size than mid-shelf and outer-shelf populations. Additionally, more individuals from the inner-shelf population reproduced after 30 days of exposure (Fig. 4A). Under reduced light levels, inner-shelf individuals that reproduced produced a substantially lower number of offspring than the population exposed to control light levels (Fig. 4B).

## Discussion

Global warming, and nutrient and sediment runoff from coastal development both exert increasing pressures on coastal coral reefs, including the Great Barrier Reef^32^,^33^. Reef-dwelling LBFs are vulnerable and consequently threatened by these pressures^25^,^34^,^35^. Our study demonstrates that exposure to reduced light levels and inorganic nutrient enriched seawater at environmentally relevant concentrations reduces the resistance of *A. lobifera* to temperature stress, leading to greater declines in survivorship and fecundity, and increased bleaching frequency and changes in chl *a* concentration. However, these responses are site-specific and varied across reef sites analysed. At a population level, this variability might be linked to genetic variation among the geographic locations we analysed, and *A. lobifera* may have the ability to produce distinct geno/phenotypes when exposed to different environments.

The number of bleached individuals was not different between light treatments, and most populations across different reef sites showed relatively low bleaching frequencies of ~25–30%, even when exposed to high temperature and nitrate levels. In general, individuals exposed to reduced light levels across reef sites showed a consistently high concentration of chl *a*. This corroborates previous results showing *A. lobifera* populations are able to quickly respond to changes in light levels^28^. Under control light conditions the addition of nitrate along with high temperature caused significant reduction in the chl *a* concentration of mid- and outer-shelf specimens, but not in their inner-shelf counterparts under the same conditions. Moreover, outer-shelf individuals also showed a slight increase in bleaching frequency under those conditions, likely associated with oxidative stress^28^,^36^. Talge and Hallock^24^ demonstrated that ultraviolet light is the main bleaching-inducing factor in *Amphistegina* sp., and that high temperature (i.e., above their natural range) coupled with intense visible light can also cause loss of symbionts. This might be the case for inner- and mid-shelf individuals, which showed a reduction in chl *a* content but consistent reproduction and low mortality. However, outer-shelf populations not only showed signs of symbiont loss, but also significant declines in survivorship and fecundity when exposed to the combined effects of heat and nitrate stress.

Survivorship was also divergent across reef sites. In the outer-shelf population, the interaction of parameters tested likely caused initial bleaching (within a few days), which quickly progressed to mortality, as evidenced by the reduction in survivorship after 30 days under control light levels. The decline in survivorship was even more severe under reduced light levels. The addition of nitrate has been demonstrated to lower the threshold of tolerance of holobiont organisms to the effects of heat stress^33^,^37^,^38^. Holobiont organisms such as corals and foraminifera restrict the access of nutrients such as nitrate and phosphate to their symbiont algae^29^,^36^. The symbiotic algae can retain their photosynthate for growth, requiring the host to utilise other food sources for respiration^16^. As a result, less carbon is translocated from the symbiont diatom to the foraminiferal host, leading to reduced storage of C in the host or test matrix and further reducing growth^31^. We demonstrated previously that the single effect of elevated temperature and dissolved nitrate caused significant reduction in antioxidant capacity and reduced growth of the host on the same outer-shelf population after 30 days^26^. Reactive oxygen species are produced in chloroplasts, mitochondria and other plastids, and when accumulated can cause oxidative stress^39^. We suggest that the mechanism responsible for a significant reduction in survivorship in outer-shelf population could be associated with oxidative stress that is not only linked to photosynthesis, but also respiration and other biochemical pathways, either in the host or symbiont^39^.

Reduced light levels completely suppressed asexual reproduction, indicating the important role of light in the reproduction of this species. *A. lobifera* tends to attain large sizes, compared to other species within the genus, thereby increasing fecundity^40^. Inner-shelf individuals clearly showed resistance to the stressors we analysed, and even though mid- and outer-shelf specimens showed somewhat successful reproduction at control levels of light, the number of offspring produced was significantly and consistently lower than in the inner-shelf population. In contrast, it is plausible that the lack of individuals reproducing in the tanks might be linked to mid- and outer-shelf populations utilising energetic resources to maintain homeostasis and growth, rather than reproduction, under stress conditions. In a natural setting, asexual reproduction is one of the main causes of mortality in adult *A. lobifera*^40^. The onset of reproduction at smaller sizes in field populations of *A. lobifera* populations is essential for survival in turbulent, shallow, subtidal environments^41^. Given that there is a scarcity of specimens larger than 1400 um in their natural environment^40^,^42^, this suggests that field individuals from the GBR undergo asexual reproduction before reaching sizes as large as the ones we observed in our experiments.

The interaction between light, temperature and nitrate can be complex. On the Great Barrier Reef, flood events typically result in a significant transitory reduction in light availability and peaks in nutrients on inshore reefs, which can occasionally reach offshore reefs^43^,^44^. Inshore reefs also experience higher temperature fluctuations than their mid-shelf and outer-shelf counterparts^45^. Our results showed that fecundity in *A. lobifera* was greatly reduced across all reef sites studied under reduced light levels. Thus, even though inner-shelf reef populations were more resistant than mid- and outer-shelf populations to elevated temperature and nitrate, they failed to consistently reproduce asexually under reduced light levels. Decreases of LBF abundance and diversity on inshore reefs of the GBR are commonly correlated to terrestrial runoff and decreases in water quality^18^,^46^,^47^. Therefore, even though inner-shelf *A. lobifera* populations can survive and reproduce under heat and nutrient stress, the reduction in light levels below their optimum is likely to be the key factor affecting their abundance and occurrence on inshore reefs of the GBR and elsewhere.

Our results demonstrate that, similar to other holobiont organisms, such as reef building corals^38^, giant clams^48^, sponges^49^ and tropical sea anemones^50^, increases in nutrient and temperature can negatively impact LBF. Local adaptation of the photo-symbionts can shape thermal tolerance of reef corals^51^, and adaptive differentiation occurs in several other marine invertebrates in response to selection imposed by strong gradients of abiotic and biotic conditions^52^. Similarly, this study suggests that phenotypic plasticity and the capacity of LBF to acclimate/adapt to their local habitat can influence the responses observed in *A. lobifera* populations collected across a water quality and temperature gradient. Whether this phenomenon is driven by adaptation of endosymbionts or the host itself remains to be investigated.

Along with reef-building corals, reef-dwelling LBFs play a fundamental role in the formation and maintenance of reef ecosystems, as they constitute a significant proportion of the reef sediment and carbonate budget^15^,^19^,^53^. Ocean warming and eutrophication associated with climate change and local impacts, respectively, are likely to have a substantial impact on the dynamics of these environments. For example, as calcification and fecundity decline so does the availability of test substrate that maintains reef islands, shifting many into a state of erosion^53^. Therefore, understanding how factors such as elevated temperature, dissolved nutrient and light interact, irrespective of the exact biochemical and physiological mechanisms, and identifying local populations that are resistant to changes in environmental conditions is vital to improving predictions of the fate (persistence versus extinction) of marine organisms under a rapidly changing climate^54^.

Coupled with results published previously^26^,^28^, these findings strongly suggest that the capacity of LBFs to acclimate to shifts in environmental conditions is influenced by their ability to regulate biochemical functions within and above their threshold of tolerance, which is shaped by their local environment. Therefore, phenotypic plasticity could be a plausible mechanism affecting the response of local populations to the environmental variation, such as elevated temperature and local nitrification. Nonetheless, improved water quality at local scales could ameliorate effects of ocean warming in LBF populations, as also demonstrated for corals^37^. Reef-dwelling LBF species, such as *Amphistegina*, have been present on reefs worldwide for over 50 million years^14^, a period encompassing a multitude of short-term and evolutionary-scale changes. Throughout these changes foraminiferal lineages have both survived and thrived, supporting the notion that many foraminiferal species might be capable of adapting to substantial ranges of environmental conditions^55^.

## Material and Methods

### Sampling collection

Pieces of dead coral rubble containing *A. lobifera* were collected from inner-, mid- and outer-shelf reefs located on the northern Great Barrier Reef in August and September 2014. Samples were collected by SCUBA divers from the back slope of reefs with similar habitat located on: (1) inner-shelf – Martin reef (14° 45′ 19.2 ″ S; 145° 20′07.9″ E); (2) mid-shelf – Lizard Island (14° 14′ 22.3″ S; 145° 27′ 58.1″ E); and (3) outer-shelf – Yonge reef (14° 35′ 50.1″ S; 145°37′ 26.3“E) at depths of 6 to 8 m (corrected to lowest astronomical tide levels)^26^. These reef sites are located along a water quality gradient and have different temperature fluctuation patterns^26^,^56^. Pieces of rubble were brought to the laboratory located at the Lizard Island Research Station (LIRS) and processed following the sampling procedures of Hallock *et al*.^22^ and Prazeres *et al*.^26^. In the laboratory, rubble pieces were scrubbed using a toothbrush, and the resultant sediment was transferred to glass Petri dishes and placed undisturbed in a flow-through aquarium system (~750 ml min^−1^). *Amphistegina* individuals were extracted and separated for the experiments. We selected adults (>0.5 mm in diameter) of uniform brown colour that displayed reticulopodial activity (indicative of good health), and specimens were acclimatised for ten days prior to commencing the experiments.

### Experimental setup

We tested the combined-effect of different levels of temperature and nutrient conditions on the foraminifera in two independent experiments exposing *A. lobifera* individuals to different light levels (control and reduced levels). We used a flow-through system (~750 ml min^−1^) supplied with raw seawater over a period of 30 days in outdoor aquaria at the LIRS. For each combined-effect experiment, we tested three different conditions of temperature and nutrients with three replicate glass tanks per treatment (n = 3), for total of 27 tanks per experiment. 100 specimens from each site were randomly assigned and placed into two separate Petri dishes in each tank (50 specimens per dish), and used to assess two important biological parameters^57^: (1) survivorship and fecundity, and (2) bleaching and chlorophyll *a* content. A total of three replicate Petri dishes per biological parameter analysed was used. These biological processes have been shown to be negatively affected by increases in temperature and nitrate levels in both laboratory and field settings^25^,^26^,^31^. Individuals of *A. lobifera* were kept in Petri dishes containing small pieces of reef rubble (<1 cm^2^).

#### Experiment 1: control light levels

Incoming natural seawater was either fed straight into the tanks (ambient controls: 24 ± 0.2 °C; mean ± SE) or stored in two different sumps, heated to either 27 ± 0.7 °C or 30 ± 0.8 °C using feedback controlled heaters, and then gravity fed into each relevant treatment tank. Nutrient levels were manipulated by adding nitrate (NO_3_^−^) to the seawater. Similar to the temperature, water was either fed straight into the tanks (ambient control: 0.09 ± 0.01 μM NO_3_^−^), or stored in sumps. Two concentrated solutions of NaNO_3_^−^ (0.1 mM and 0.4 mM) were delivered into 5 l sumps at a constant rate (1 ml min^−1^) using peristaltic pumps. Water from the sumps was distributed into each of the respective treatment tanks at 150 ml min^−^, and NO_3_^−^ concentrations in the tanks were kept at either 1.1 ± 0.3 or 3.2 ± 0.9 μM NO_3_^−^. Tanks were kept in the outdoor aquaria under shade cloth reducing natural sunlight light levels down to 98.7 ± 8.2 μmol photon m^−2^ s^−1^ at midday.

#### Experiment 2: reduced light levels

The same system as experiment 1 was used for experiment 2, in which water was delivered straight into the tanks (control treatments), and temperature and nutrient manipulations were regulated in the overhead sumps and then fed into the relevant treatment tanks. Temperature was kept constant at 24 ± 0.3 °C (ambient controls), or heated up to 27 ± 0.8 °C or 30 ± 0.2 °C. Nitrate levels in ambient control tanks were 0.07 ± 0.01 μM NO_3_^−^, or 1.2 ± 0.4 and 3.8 ± 0.7 μM NO_3_^−^. Tanks were kept in the same outdoor aquaria used in experiment 1 under shade cloth reducing natural sunlight light levels down to 30.5 ± 2.9 μmol photon m^−2^ s^−1^ at midday.

For both experiment 1 and 2, HOBO^®^ data loggers (Model UA-002-64) were placed in each sump, and in one tank per treatment to record temperature and light intensity every 15 min, for the duration of the experiments. Additionally, two replicate water samples were collected from each tank every week, filtered (0.45 μm) and frozen for further laboratory analysis. Dissolved nitrate concentrations were determined using a Lachat flow injection analyser in the Advanced Water Management Centre at the University of Queensland (Brisbane, Australia).

### Survivorship and fecundity assessment

A total number of 50 individuals from experiments 1 and 2 were assessed daily regarding the number of dead individuals and reproduction. Using a stereomicroscope, dead individuals were counted and percentage calculated. Dead individuals were adult-sized (from 0.5 mm to 1.2 mm of diameter), had no obvious reticulopodial activity, were usually epiphytised and contained no trace of internal debris.

Fecundity was calculated according to Hallock^40^. *Amphistegina* sp. present a trimorphic life cycle, which consists of alternations between gamont, agamont and schizont forms^58^,^59^. The gamont is the haploid form, which produces gametes and is linked to sexual reproduction. The schizont and agamont forms are diploids, and are produced by multiple fission or asexual reproduction. Only asexual reproduction was observed in the tanks during the experiment. Empty tests that underwent reproduction were counted. As opposed to dead tests, individuals that underwent asexual reproduction are bigger than normal adults (>1.2 mm diameter, with abnormal large proloculus), have a broken/dissolved last chamber and newborns around their tests. To calculate fecundity, number of individuals that reproduced were counted, and summed at the end of each experiment. Total number of offspring produced per individual, and adult diameter was noted^40^. Age of individuals that underwent reproduction could not be determined as individuals were collected from their natural environment at adult sizes.

### Bleaching frequency and chlorophyll a analysis

For both experiments 1 and 2, we determined the frequency of bleaching by counting the number of individuals showing any sign of symbiont loss (ranging from small white spots to extensive white or “mottled” areas) and the percentage of bleached individuals was calculated. Bleached individuals were easily identified as they usually contain some remanent of cytoplasm, resulting from the digestion of diatoms^24^. Chlorophyll *a* (Chl *a*) content of live individuals of *A. lobifera* holobionts was also measured. Each specimen was individually placed into 1.5 ml vials filled with 320 μl of 90% ethanol to extract pigment^25^,^60^. Samples were extracted in the dark for 24 h at 4 °C. 200 ul of the extraction liquid was taken and absorbance at 630, 663 and 750 nm was measured using a microplate reader (SpectraMax^®^ Plus 384, Molecular Devices, Australia). After extraction, specimens were dried at room temperature for 24 h, and weighed using a very fine scale (Mettler Toledo XS105, Australia). Chl *a* concentration was calculated according to Hosono *et al*.^60^, and normalised using test weight.

### Data analyses

Each combined-effect experiment was analysed separately, as they represent two independent experiments. Survivorship and bleaching frequency were analysed with a Generalised Linear Mixed Model (GLMM) using the package *lme4* in R^61^. As these parameters represent percentages, we used a binomial GLMM^62^ to determine differences between experimental conditions among reef sites. Chlorophyll *a* (Chl *a*) content was analysed with a Partly Nested ANOVA using the function *aov* in R. Because the Chl *a* content at control light levels violated the homogeneity of variance assumption, data were square root transformed. In all cases, reef site, temperature and nitrate concentration were considered fixed factors, while tank was the random effect. Tank was considered the blocking factor, and temperature and nitrate between-block effects. Reef site was used as a within-block effect. Significance of single and interaction effects of fixed factors was quantified using Type III Sum of Squares. Tukey’s *post-hoc* test was conducted using the package *multcomp* in R. Fecundity was analysed using an unbalanced ANOVA to test for differences in number of new individuals produced by adults exposed to different treatments across reef sites. A linear regression using the total number of new individuals produced and size of adults that reproduced in each reef site population at any given treatment was plotted. Due to the small sample size of overall outer-shelf individuals that reproduced, assumptions of normality and homogeneity of variance were not met. Therefore, we performed a non-parametric Kruskal-Wallis test to detect differences in the number of offspring between treatments, instead of an unbalanced ANOVA. A non-parametric (Kendall’s) robust regression using the package *mblm* in R was performed to assess the correlation between test size and number of individuals produced by outer-shelf *A. lobifera* exposed to different treatments. Regression slope coefficients of the linearised function for each reef site population, which defines the relationship between number of offspring and size of adult individuals from each reef site population, were compared using a Student’s *t*-test. In all cases, assessment of normality and homogeneity of variance were carried out using Shapiro-Wilk’s and Levene’s tests, respectively.

## Additional Information

**How to cite this article:** Prazeres, M. *et al*. Variation in sensitivity of large benthic Foraminifera to the combined effects of ocean warming and local impacts. *Sci. Rep.* **7**, 45227; doi: 10.1038/srep45227 (2017).

**Publisher's note:** Springer Nature remains neutral with regard to jurisdictional claims in published maps and institutional affiliations.

## Supplementary Material

Supplementary Material

## Figures and Tables

**Table 1:** 

Response variable	df	χ^2^	P-value
Reef site	2	0.0001	0.99
Temperature	2	19.8421	**<0.001**
Nitrate	2	25.8586	**<0.001**
Reef site*Temperature	4	4.8325	0.30
Reef site*Nitrate	4	14.888	**0.005**
Temperature*Nitrate	4	31.6030	**<0.001**
Reef site*Temperature*Nitrate	8	25.4276	**0.002**

Generalised Linear Mixed Model results of survivorship in *Amphistegina lobifera* populations collected from different reef sites and exposed to different conditions of temperature*nitrate, under control light levels.

**Table 2:** 

Response variable	df	χ^2^	P-value
Reef site	2	5.93	**0.05**
Temperature	2	105.06	**<0.001**
Nitrate	2	27.02	0.09
Reef site*Temperature	4	27.80	**<0.001**
Reef site*Nitrate	4	23.36	**<0.001**
Temperature*Nitrate	4	29.09	**<0.001**
Reef site*Temperature*Nitrate	8	38.17	**<0.001**

Generalised Linear Mixed Model results of bleaching frequency in *Amphistegina lobifera* populations collected from different reef sites and exposed to different conditions of temperature*nitrate, under control light levels.

**Table 3:** 

Response variable	df	MS	F-value	P-value
Reef site	2	84.61	3.646	**0.03**
Reef site*Temperature	4	22.52	0.970	0.42
Reef site*Nitrate	4	7.96	0.343	0.84
Reef site*Temperature*Nitrate	8	45.39	1.956	**0.05**
Residuals	216	23.20		

Mixed-model ANOVA results for chlorophyll *a* concentration of *Amphistegina lobifera* collected from different reef sites, and exposed to different conditions of temperature*nitrate, under control light levels.

**Table 4:** 

Response variable	df	χ^2^	P-value
Reef site	2	0.0001	1.00
Temperature	2	0.0001	0.99
Nitrate	2	0.0001	0.99
Reef site*Temperature	4	27.7278	**<0.001**
Reef site*Nitrate	4	0.0001	1.00
Temperature*Nitrate	4	8.32	0.08
Reef site*Temperature*Nitrate	8	49.7868	**<0.001**

Generalised Linear Mixed Model results of survivorship in *Amphistegina lobifera* populations collected from different reef sites and exposed to different conditions of temperature*nitrate, under low light levels.

**Table 5:** 

Response variable	df	χ^2^	P-value
Reef site	2	0.1 × 10^−3^	0.99
Temperature	2	0.16	0.92
Nitrate	2	1.21	0.54
Reef site*Temperature	4	10.41	**0.03**
Reef site*Nitrate	4	2.36	0.66
Temperature*Nitrate	4	3.19	0.52
Reef site*Temperature*Nitrate	8	11.11	0.19

Generalised Linear Mixed Model results of bleaching frequency in *Amphistegina lobifera* populations collected from different reef sites and exposed to different conditions of temperature*nitrate, under low light levels.

**Table 6:** 

Response variable	df	MS	F-value	P-value
Reef site	2	83051	5.315	**<0.01**
Reef site*Temperature	4	47050	3.013	**0.01**
Reef site*Nitrate	4	7719	0.494	0.73
Reef site*Temperature*Nitrate	8	20054	1.284	0.25
Residuals	215	15618		

Mixed-model ANOVA results for chlorophyll *a* concentration of *Amphistegina lobifera* collected from different reef sites, and exposed to different conditions of temperature*nitrate, under low light levels.

**Table 7:** Comparison between slopes among fecundity of *A. lobifera* collected from different reef sites across all temperature*nitrate treatments.

Comparison between slopes	df	SSD	t-test	P-value
Inner-shelf x Mid-shelf	41	0.24	3.51	**0.001**
Inner-shelf x Outer-shelf	31	0.22	6.13	**<0.001**
Mid-shelf x Outer-shelf	22	0.21	2.73	**0.02**
Inner-shelf (Control) x Inner-shelf (Low)	30	0.56	3.31	**0.002**

SDD stands for the standard error of the difference between slopes of the linearised function defining the relationship between shell size (mm^2^) and number of individuals produced.
