# Supplementary material for: Variation in sensitivity of large benthic Foraminifera to the combined effects of ocean warming and local impacts
Source: Sci Rep. 2017 Mar 23;7:45227. doi: 10.1038/srep45227 (PMC5362903; doi:10.1038/srep45227)
Supplement: Supplementary Material [file srep45227-s1.doc]

**Supplementary material**

**Variation in sensitivity of large benthic Foraminifera to the combined effects of ocean warming and local impacts**

Martina Prazeres^1,*^, T. Edward Roberts^2^, John M. Pandolfi^1^

^1^ ARC Centre of Excellent for Coral Reef Studies and School of Biological Sciences, The University of Queensland, St. Lucia, QLD 4072, Australia

^2^ ARC Centre of Excellent for Coral Reef Studies, James Cook University, Townsville, QLD 4811, Australia

**Table S1.** Reproduction rates of *A. lobifera* collected from different reef sites and exposed to different temperature*nitrate treatments after 30 days under control or low light conditions. T1, T2 and T3 stand for 24 °C, 27 °C and 30 °C, respectively. N1, N2 and N3 represent 0.09, 1.5 and 3.2 μM NO_3_^-^, respectively.

| **Reef site** | **Treatment** | **Total N** | **Reproduction** | **%** |
| --- | --- | --- | --- | --- |
| Inner-shelf (control light level) | T1*N1 | 150 | 5 | 3.33 |
|  | T1*N2 | 150 | 1 | 0.67 |
|  | T1*N3 | 150 | 0 | 0.00 |
|  | T2*N1 | 150 | 5 | 3.33 |
|  | T2*N2 | 150 | 4 | 2.67 |
|  | T2*N3 | 150 | 6 | 4.00 |
|  | T3*N1 | 150 | 3 | 2.00 |
|  | T3*N2 | 150 | 3 | 2.00 |
|  | T3*N3 | 150 | 2 | 1.33 |
| TOTAL |  |  |  | **19.33** |
| Inner-shelf (low light level) | T1*N1 | 150 | 7 | 4.66 |
| TOTAL |  |  |  | **4.66** |
| Mid-shelf (control light level) | T1*N1 | 150 | 1 | 0.67 |
|  | T1*N2 | 150 | 5 | 3.33 |
|  | T1*N3 | 150 | 4 | 2.67 |
|  | T2*N1 | 150 | 1 | 0.67 |
|  | T2*N2 | 150 | 1 | 0.67 |
|  | T2*N3 | 150 | 3 | 2.00 |
|  | T3*N1 | 150 | 1 | 0.67 |
|  | T3*N2 | 150 | 1 | 0.67 |
|  | T3*N3 | 150 | 1 | 0.67 |
| TOTAL |  |  |  | **12.00** |
| Outer-shelf  (control light level) | T1*N1 | 150 | 2 | 1.33 |
|  | T1*N2 | 150 | 0 | 0.00 |
|  | T1*N3 | 150 | 2 | 1.33 |
|  | T2*N1 | 150 | 0 | 0.00 |
|  | T2*N2 | 150 | 1 | 0.67 |
|  | T2*N3 | 150 | 0 | 0.00 |
|  | T3*N1 | 150 | 1 | 0.67 |
|  | T3*N2 | 150 | 1 | 0.67 |
|  | T3*N3 | 150 | 1 | 0.67 |
| TOTAL |  |  |  | **5.33** |

**Table S2**. Tukey’s HSD *post hoc* test of the pairwise comparisons of survivorship among populations collected from inner-, mid- and outer-shelf reefs exposed to different *temperature*nitrate* conditions under control light levels. The bold numbers indicated significant pairwise comparisons (P-value < 0.05). T1, T2 and T3 represents temperature treatments of 24 °C, 27 °C and 30°C, respectively. N1, N2 and N3 stand for 0.09 uM, 1.5 and 3.2 uM NO_3_^1-^, respectively.

| CONTROL LIGHT | | | Inner-shelf | Inner-shelf | Inner-shelf | Inner-shelf | Inner-shelf | Inner-shelf | Inner-shelf | Inner-shelf | Inner-shelf | Mid-shelf | Mid-shelf | Mid-shelf | Mid-shelf | Mid-shelf | Mid-shelf | Mid-shelf | Mid-shelf | Mid-shelf | Outer-shelf | Outer-shelf | Outer-shelf | Outer-shelf | Outer-shelf | Outer-shelf | Outer-shelf | Outer-shelf | Outer-shelf |
| --- | --- | --- | --- | --- | --- | --- | --- | --- | --- | --- | --- | --- | --- | --- | --- | --- | --- | --- | --- | --- | --- | --- | --- | --- | --- | --- | --- | --- | --- |
|  |  |  | T1 | T1 | T1 | T2 | T2 | T2 | T3 | T3 | T3 | T1 | T1 | T1 | T2 | T2 | T2 | T3 | T3 | T3 | T1 | T1 | T1 | T2 | T2 | T2 | T3 | T3 | T3 |
|  |  |  | N1 | N2 | N3 | N1 | N2 | N3 | N1 | N2 | N3 | N1 | N2 | N3 | N1 | N2 | N3 | N1 | N2 | N3 | N1 | N2 | N3 | N1 | N2 | N3 | N1 | N2 | N3 |
| Inner-shelf | T1 | N1 |  |  |  |  |  |  |  |  |  |  |  |  |  |  |  |  |  |  |  |  |  |  |  |  |  |  |  |
| Inner-shelf | T1 | N2 | 1.00 |  |  |  |  |  |  |  |  |  |  |  |  |  |  |  |  |  |  |  |  |  |  |  |  |  |  |
| Inner-shelf | T1 | N3 | 1.00 | 1.00 |  |  |  |  |  |  |  |  |  |  |  |  |  |  |  |  |  |  |  |  |  |  |  |  |  |
| Inner-shelf | T2 | N1 | 1.00 | 1.00 | 1.00 |  |  |  |  |  |  |  |  |  |  |  |  |  |  |  |  |  |  |  |  |  |  |  |  |
| Inner-shelf | T2 | N2 | 1.00 | 0.528 | 0.99 | 0.97 |  |  |  |  |  |  |  |  |  |  |  |  |  |  |  |  |  |  |  |  |  |  |  |
| Inner-shelf | T2 | N3 | 1.00 | **<0.1** | **<0.1** | **<0.1** | 0.66 |  |  |  |  |  |  |  |  |  |  |  |  |  |  |  |  |  |  |  |  |  |  |
| Inner-shelf | T3 | N1 | 1.00 | 1.00 | 1.00 | 1.00 | 0.99 | **<0.1** |  |  |  |  |  |  |  |  |  |  |  |  |  |  |  |  |  |  |  |  |  |
| Inner-shelf | T3 | N2 | 1.00 | **<0.1** | 0.44 | 0.32 | 1.00 | 1.0 | 0.58 |  |  |  |  |  |  |  |  |  |  |  |  |  |  |  |  |  |  |  |  |
| Inner-shelf | T3 | N3 | 1.00 | **<0.1** | 0.35 | 0.25 | 1.00 | 1.00 | 0.48 | 1.00 |  |  |  |  |  |  |  |  |  |  |  |  |  |  |  |  |  |  |  |
| Mid-shelf | T1 | N1 | 1.00 | 1.00 | 1.00 | 1.00 | 1.00 | 1.00 | 1.00 | 1.00 | 1.00 |  |  |  |  |  |  |  |  |  |  |  |  |  |  |  |  |  |  |
| Mid-shelf | T1 | N2 | 1.00 | 0.637 | 0.99 | 0.98 | 1.00 | 0.54 | 1.00 | 1.00 | 0.99 | 1.00 |  |  |  |  |  |  |  |  |  |  |  |  |  |  |  |  |  |
| Mid-shelf | T1 | N3 | 1.00 | 0.984 | 1.00 | 1.00 | 1.00 | 0.11 | 1.00 | 0.91 | 0.85 | 1.00 | 1.00 |  |  |  |  |  |  |  |  |  |  |  |  |  |  |  |  |
| Mid-shelf | T2 | N1 | 1.00 | 1.00 | 1.00 | 1.00 | 0.53 | **<0.1** | 1.00 | **<0.1** | **<0.1** | 1.00 | 0.63 | 0.98 |  |  |  |  |  |  |  |  |  |  |  |  |  |  |  |
| Mid-shelf | T2 | N2 | 1.00 | **<0.1** | 0.27 | 0.19 | 1.00 | 1.00 | 0.39 | 1.00 | 1.00 | 1.00 | 0.99 | 0.77 | **<0.1** |  |  |  |  |  |  |  |  |  |  |  |  |  |  |
| Mid-shelf | T2 | N3 | 1.00 | 0.194 | 0.83 | 0.71 | 1.00 | 0.96 | 0.92 | 1.00 | 1.00 | 1.00 | 1.00 | 0.99 | 0.19 | 1.00 |  |  |  |  |  |  |  |  |  |  |  |  |  |
| Mid-shelf | T3 | N1 | 1.00 | **<0.1** | **<0.1** | **<0.1** | 0.19 | 1.00 | **<0.1** | 0.92 | 0.96 | 1.00 | 0.13 | **<0.1** | **<0.1** | 0.98 | 0.57 |  |  |  |  |  |  |  |  |  |  |  |  |
| Mid-shelf | T3 | N2 | 1.00 | **<0.1** | **<0.1** | **<0.1** | 0.19 | 1.00 | **<0.1** | 0.93 | 0.96 | 1.00 | 0.13 | **<0.1** | **<0.1** | 0.98 | 0.58 | 1.00 |  |  |  |  |  |  |  |  |  |  |  |
| Mid-shelf | T3 | N3 | 1.00 | 1.00 | 1.00 | 1.00 | **<0.**1 | 0.23 | 1.00 | **<0.1** | **<0.1** | 1.00 | 1.00 | 1.00 | 1.00 | **<0.1** | **<0.1** | 0.72 | 0.72 |  |  |  |  |  |  |  |  |  |  |
| Outer-shelf | T1 | N1 | 1.00 | 1.00 | 1.00 | 1.00 | 0.82 | **<0.1** | 1.00 | 0.14 | 0.11 | 1.00 | 0.89 | 1.00 | 1.00 | **<0.1** | 0.42 | **<0.1** | **<0.1** | 1.00 |  |  |  |  |  |  |  |  |  |
| Outer-shelf | T1 | N2 | 1.00 | 1.00 | 1.00 | 1.00 | 0.92 | **<0.1** | 1.00 | 0.22 | 0.16 | 1.00 | 0.96 | 1.00 | 1.00 | 0.12 | 0.56 | **<0.1** | **<0.1** | 1.00 | 1.00 |  |  |  |  |  |  |  |  |
| Outer-shelf | T1 | N3 | 1.00 | 0.99 | 1.00 | 1.00 | 1.00 | **<0.1** | 1.00 | 0.72 | 0.62 | 1.00 | 1.00 | 1.00 | 0.99 | 0.52 | 0.96 | **<0.1** | **<0.1** | 1.00 | 1.00 | 1.00 |  |  |  |  |  |  |  |
| Outer-shelf | T2 | N1 | 1.00 | 1.00 | 1.00 | 1.00 | 1.00 | 1.00 | 1.00 | 1.00 | 1.00 | 1.00 | 1.00 | 1.00 | 1.00 | 1.00 | 1.00 | 1.00 | 1.00 | 1.00 | 1.00 | 1.00 | 1.00 |  |  |  |  |  |  |
| Outer-shelf | T2 | N2 | 1.00 | **<0.1** | 0.21 | 0.14 | 0.99 | 1.00 | 0.31 | 1.00 | 1.00 | 1.00 | 0.99 | 0.68 | **<0.1** | 1.00 | 1.00 | 0.99 | 0.99 | **<0.1** | **<0.1** | **<0.1** | 0.42 | 1.00 |  |  |  |  |  |
| Outer-shelf | T2 | N3 | 1.00 | **<0.1** | **<0.1** | **<0.1** | 0.14 | 1.00 | **<0.1** | 0.87 | 0.93 | 1.00 | **<0.1** | **<0.1** | **<0.1** | 0.96 | 0.48 | 1.00 | 1.00 | 0.81 | **<0.1** | **<0.1** | **<0.1** | 1.00 | 0.98 |  |  |  |  |
| Outer-shelf | T3 | N1 | 1.00 | 1.00 | 1.00 | 1.00 | 1.00 | **<0.1** | 1.00 | 1.00 | 1.00 | 1.00 | 1.00 | 1.00 | 1.00 | 1.00 | 1.00 | **<0.1** | **<0.1** | 0.81 | 1.00 | 1.00 | 1.00 | 1.00 | 1.00 | **<0.1** |  |  |  |
| Outer-shelf | T3 | N2 | 1.00 | 1.00 | 1.00 | 1.00 | 1.00 | 1.00 | 1.00 | 1.00 | 1.00 | 1.00 | 1.00 | 1.00 | 1.00 | 1.00 | 1.00 | 1.00 | 1.00 | 1.00 | 1.00 | 1.00 | 1.00 | 1.00 | 1.00 | 1.00 | **<0.1** |  |  |
| Outer-shelf | T3 | N3 | 1.00 | 1.00 | 1.00 | 1.00 | 1.00 | 1.00 | 1.00 | 1.00 | 1.00 | 1.00 | **1.00** | **1.00** | 1.00 | 1.00 | 1.00 | 1.00 | 1.00 | 1.00 | 1.00 | 1.00 | 1.00 | 1.00 | 1.00 | 1.00 | 1.00 | **<0.1** |  |

**Table S3**. Tukey’s HSD *post hoc* test of the pairwise comparisons of **bleaching frequency** among populations collected from inner-, mid- and outer-shelf reefs exposed to different *temperature*nitrate* conditions under **control light levels**. The bold numbers indicated significant pairwise comparisons (P-value < 0.05). T1, T2 and T3 represents temperature treatments of 24 °C, 27 °C and 30°C, respectively. N1, N2 and N3 stand for 0.09 uM, 1.5 and 3.2 uM NO_3_^1-^, respectively.

| CONTROL LIGHT | | | Inner-shelf | Inner-shelf | Inner-shelf | Inner-shelf | Inner-shelf | Inner-shelf | Inner-shelf | Inner-shelf | Inner-shelf | Mid-shelf | Mid-shelf | Mid-shelf | Mid-shelf | Mid-shelf | Mid-shelf | Mid-shelf | Mid-shelf | Mid-shelf | Outer-shelf | Outer-shelf | Outer-shelf | Outer-shelf | Outer-shelf | Outer-shelf | Outer-shelf | Outer-shelf | Outer-shelf |
| --- | --- | --- | --- | --- | --- | --- | --- | --- | --- | --- | --- | --- | --- | --- | --- | --- | --- | --- | --- | --- | --- | --- | --- | --- | --- | --- | --- | --- | --- |
|  |  |  | T1 | T1 | T1 | T2 | T2 | T2 | T3 | T3 | T3 | T1 | T1 | T1 | T2 | T2 | T2 | T3 | T3 | T3 | T1 | T1 | T1 | T2 | T2 | T2 | T3 | T3 | T3 |
|  |  |  | N1 | N2 | N3 | N1 | N2 | N3 | N1 | N2 | N3 | N1 | N2 | N3 | N1 | N2 | N3 | N1 | N2 | N3 | N1 | N2 | N3 | N1 | N2 | N3 | N1 | N2 | N3 |
| Inner-shelf | T1 | N1 |  |  |  |  |  |  |  |  |  |  |  |  |  |  |  |  |  |  |  |  |  |  |  |  |  |  |  |
| Inner-shelf | T1 | N2 | 1.00 |  |  |  |  |  |  |  |  |  |  |  |  |  |  |  |  |  |  |  |  |  |  |  |  |  |  |
| Inner-shelf | T1 | N3 | 1.00 | 1.00 |  |  |  |  |  |  |  |  |  |  |  |  |  |  |  |  |  |  |  |  |  |  |  |  |  |
| Inner-shelf | T2 | N1 | 0.88 | 1.00 | 0.71 |  |  |  |  |  |  |  |  |  |  |  |  |  |  |  |  |  |  |  |  |  |  |  |  |
| Inner-shelf | T2 | N2 | 1.00 | 1.00 | **<0.01** | 0.51 |  |  |  |  |  |  |  |  |  |  |  |  |  |  |  |  |  |  |  |  |  |  |  |
| Inner-shelf | T2 | N3 | 0.28 | 1.00 | **<0.01** | **<0.01** | 0.99 |  |  |  |  |  |  |  |  |  |  |  |  |  |  |  |  |  |  |  |  |  |  |
| Inner-shelf | T3 | N1 | **<0.01** | 1.00 | **<0.01** | 0.01 | 0.99 | **1.00** |  |  |  |  |  |  |  |  |  |  |  |  |  |  |  |  |  |  |  |  |  |
| Inner-shelf | T3 | N2 | **<0.01** | 1.00 | **<0.01** | 0.85 | 1.00 | 0.87 | 0.95 |  |  |  |  |  |  |  |  |  |  |  |  |  |  |  |  |  |  |  |  |
| Inner-shelf | T3 | N3 | 0.64 | 1.00 | **0.02** | 0.99 | 1.00 | 0.52 | 0.69 | 1.00 |  |  |  |  |  |  |  |  |  |  |  |  |  |  |  |  |  |  |  |
| Mid-shelf | T1 | N1 | 0.93 | 1.00 | 0.60 | 1.00 | 0.63 | **<0.01** | **0.01** | 0.92 | 0.99 |  |  |  |  |  |  |  |  |  |  |  |  |  |  |  |  |  |  |
| Mid-shelf | T1 | N2 | 1.00 | 1.00 | **<0.01** | 0.51 | 1.00 | 0.99 | 0.99 | 1.00 | 1.00 | 0.63 |  |  |  |  |  |  |  |  |  |  |  |  |  |  |  |  |  |
| Mid-shelf | T1 | N3 | 0.28 | 1.00 | **<0.01** | 0.01 | 0.99 | 1.00 | 1.00 | 0.95 | 0.69 | **0.01** | 0.99 |  |  |  |  |  |  |  |  |  |  |  |  |  |  |  |  |
| Mid-shelf | T2 | N1 | **<0.01** | 1.00 | 1.00 | 0.39 | <0.01 | **<0.01** | **<0.01** | **<0.01** | **0.01** | 0.31 | **<0.01** | **<0.01** |  |  |  |  |  |  |  |  |  |  |  |  |  |  |  |
| Mid-shelf | T2 | N2 | 0.59 | 1.00 | 0.60 | 1.00 | 0.63 | **<0.01** | **0.01** | 0.92 | 0.99 | 1.00 | 0.62 | **0.01** | 0.31 |  |  |  |  |  |  |  |  |  |  |  |  |  |  |
| Mid-shelf | T2 | N3 | 1.00 | 1.00 | **<0.01** | 0.08 | 1.00 | 1.00 | 1.00 | 0.99 | 0.98 | 0.12 | 1.00 | 1.00 | **<0.01** | 0.12 |  |  |  |  |  |  |  |  |  |  |  |  |  |
| Mid-shelf | T3 | N1 | **0.03** | 1.00 | **<0.01** | **0.78** | 1.00 | 0.93 | 0.98 | 1.00 | 1.00 | 0.87 | 1.00 | 0.97 | **<0.01** | 0.87 | 1.00 |  |  |  |  |  |  |  |  |  |  |  |  |
| Mid-shelf | T3 | N2 | 0.54 | 1.00 | 0.70 | **1.00** | 0.51 | **<0.01** | **0.01** | 0.85 | 0.99 | 1.00 | 0.51 | **<0.01** | 0.39 | 1.00 | 0.08 | 0.78 |  |  |  |  |  |  |  |  |  |  |  |
| Mid-shelf | T3 | N3 | 1.00 | 1.00 | 0.23 | 1.00 | 0.95 | 0.06 | 0.11 | 0.99 | 1.00 | 1.00 | 0.95 | 0.11 | 0.11 | 1.00 | **0.46** | 0.99 | 1.00 |  |  |  |  |  |  |  |  |  |  |
| Outer-shelf | T1 | N1 | 1.00 | 1.00 | 1.00 | 0.84 | <0.01 | **<0.01** | **<0.01** | **0.01** | **0.04** | 0.75 | **<0.01** | **<0.01** | 1.00 | 0.75 | <0.01 | **<0.01** | 0.84 | 0.35 |  |  |  |  |  |  |  |  |  |
| Outer-shelf | T1 | N2 | 0.96 | 1.00 | **<0.01** | 0.61 | 1.00 | 0.98 | 0.99 | 1.00 | 1.00 | 0.75 | 1.00 | 0.99 | **<0.01** | 0.72 | 1.00 | 1.00 | 0.61 | 0.97 | **<0.01** |  |  |  |  |  |  |  |  |
| Outer-shelf | T1 | N3 | 0.36 | 1.00 | 0.07 | 0.99 | 0.99 | 0.24 | 0.38 | 1.00 | 1.00 | 1.00 | 0.99 | 0.38 | **0.03** | 1.00 | 0.86 | 1.00 | 0.99 | 1.00 | 0.11 | 0.99 |  |  |  |  |  |  |  |
| Outer-shelf | T2 | N1 | 0.99 | 1.00 | **<0.01** | 0.60 | 1.00 | 0.98 | 0.99 | 1.00 | 1.00 | 0.72 | 1.00 | 0.99 | **<0.01** | 0.72 | 1.00 | 1.00 | 0.60 | 0.97 | **<0.01** | 1.00 | 0.99 |  |  |  |  |  |  |
| Outer-shelf | T2 | N2 | 0.36 | 1.00 | **<0.01** | 0.78 | 1.00 | 0.93 | 0.97 | 1.00 | 1.00 | 0.87 | 1.00 | 0.98 | **<0.01** | 0.87 | 1.00 | 1.00 | 0.78 | 0.99 | **<0.01** | 1.00 | 1.00 | 1.00 |  |  |  |  |  |
| Outer-shelf | T2 | N3 | 0.54 | 1.00 | **<0.01** | **<0.01** | 0.98 | 1.00 | 1.00 | 0.82 | 0.43 | **<0.01** | 0.98 | 1.00 | **<0.01** | **<0.01** | 1.00 | 0.88 | **<0.01** | **0.04** | **<0.01** | 0.96 | 0.18 | 0.96 | 0.88 |  |  |  |  |
| Outer-shelf | T3 | N1 | **<0.01** | 1.00 | **<0.01** | 0.02 | 0.99 | 1.00 | 1.00 | 0.98 | 0.83 | **0.03** | 0.99 | 1.00 | **<0.01** | **0.03** | 1.00 | 0.99 | **0.02** | 0.18 | **<0.01** | 0.99 | 0.54 | 0.99 | 0.99 | 1.00 |  |  |  |
| Outer-shelf | T3 | N2 | **<0.01** | 1.00 | **<0.01** | <0.01 | 0.07 | 0.98 | 0.93 | **0.01** | **<0.01** | **<0.01** | 0.07 | 0.93 | **<0.01** | **<0.01** | 0.50 | **0.02** | **<0.01** | **<0.01** | **<0.01** | 0.05 | **<0.01** | 0.05 | **0.02** | 0.99 | 0.83 |  |  |
| Outer-shelf | T3 | N3 | **<0.01** | 1.00 | 1.00 | 1.00 | 1.00 | 1.00 | 1.00 | 1.00 | 1.00 | 1.00 | 1.00 | 1.00 | 1.00 | 1.00 | 1.00 | 1.00 | 1.00 | 1.00 | 1.00 | 1.00 | 1.00 | 1.00 | 1.00 | 1.00 | 1.00 | 1.00 |  |

**Table S4**. Tukey’s HSD *post hoc* test of the pairwise comparisons of **chlorophyll *a*** among populations collected from inner-, mid- and outer-shelf reefs exposed to different *temperature*nitrate* conditions under **control light levels**. The bold numbers indicated significant pairwise comparisons (P-value < 0.05). T1, T2 and T3 represents temperature treatments of 24 °C, 27 °C and 30°C, respectively. N1, N2 and N3 stand for 0.09 uM, 1.5 and 3.2 uM NO_3_^1-^, respectively.

| CONTROL LIGHT | | | Inner-shelf | Inner-shelf | Inner-shelf | Inner-shelf | Inner-shelf | Inner-shelf | Inner-shelf | Inner-shelf | Inner-shelf | Mid-shelf | Mid-shelf | Mid-shelf | Mid-shelf | Mid-shelf | Mid-shelf | Mid-shelf | Mid-shelf | Mid-shelf | Outer-shelf | Outer-shelf | Outer-shelf | Outer-shelf | Outer-shelf | Outer-shelf | Outer-shelf | Outer-shelf | Outer-shelf |
| --- | --- | --- | --- | --- | --- | --- | --- | --- | --- | --- | --- | --- | --- | --- | --- | --- | --- | --- | --- | --- | --- | --- | --- | --- | --- | --- | --- | --- | --- |
|  |  |  | T1 | T1 | T1 | T2 | T2 | T2 | T3 | T3 | T3 | T1 | T1 | T1 | T2 | T2 | T2 | T3 | T3 | T3 | T1 | T1 | T1 | T2 | T2 | T2 | T3 | T3 | T3 |
|  |  |  | N1 | N2 | N3 | N1 | N2 | N3 | N1 | N2 | N3 | N1 | N2 | N3 | N1 | N2 | N3 | N1 | N2 | N3 | N1 | N2 | N3 | N1 | N2 | N3 | N1 | N2 | N3 |
| Inner-shelf | T1 | N1 |  |  |  |  |  |  |  |  |  |  |  |  |  |  |  |  |  |  |  |  |  |  |  |  |  |  |  |
| Inner-shelf | T1 | N2 | 0.99 |  |  |  |  |  |  |  |  |  |  |  |  |  |  |  |  |  |  |  |  |  |  |  |  |  |  |
| Inner-shelf | T1 | N3 | 0.88 | 0.99 |  |  |  |  |  |  |  |  |  |  |  |  |  |  |  |  |  |  |  |  |  |  |  |  |  |
| Inner-shelf | T2 | N1 | 1.00 | 1.00 | 0.99 |  |  |  |  |  |  |  |  |  |  |  |  |  |  |  |  |  |  |  |  |  |  |  |  |
| Inner-shelf | T2 | N2 | 0.93 | 0.99 | 1.00 | 0.99 |  |  |  |  |  |  |  |  |  |  |  |  |  |  |  |  |  |  |  |  |  |  |  |
| Inner-shelf | T2 | N3 | 1.00 | 1.00 | 0.93 | 1.00 | 0.96 |  |  |  |  |  |  |  |  |  |  |  |  |  |  |  |  |  |  |  |  |  |  |
| Inner-shelf | T3 | N1 | 1.00 | 1.00 | 0.98 | 1.00 | 0.99 | 1.00 |  |  |  |  |  |  |  |  |  |  |  |  |  |  |  |  |  |  |  |  |  |
| Inner-shelf | T3 | N2 | 1.00 | 0.99 | 0.75 | 1.00 | 0.83 | 1.00 | 1.00 |  |  |  |  |  |  |  |  |  |  |  |  |  |  |  |  |  |  |  |  |
| Inner-shelf | T3 | N3 | 0.97 | 0.39 | **0.01** | 0.62 | **0.01** | 0.93 | 0.81 | 0.99 |  |  |  |  |  |  |  |  |  |  |  |  |  |  |  |  |  |  |  |
| Mid-shelf | T1 | N1 | 0.99 | 1.00 | 1.00 | 1.00 | 1.00 | 0.99 | 0.99 | 0.99 | 0.16 |  |  |  |  |  |  |  |  |  |  |  |  |  |  |  |  |  |  |
| Mid-shelf | T1 | N2 | 0.99 | 1.00 | 0.99 | 1.00 | 0.99 | 1.00 | 1.00 | 0.99 | 0.35 | 1.00 |  |  |  |  |  |  |  |  |  |  |  |  |  |  |  |  |  |
| Mid-shelf | T1 | N3 | 1.00 | 1.00 | 0.99 | 1.00 | 0.99 | 1.00 | 1.00 | 1.00 | 0.67 | 1.00 | 1.00 |  |  |  |  |  |  |  |  |  |  |  |  |  |  |  |  |
| Mid-shelf | T2 | N1 | 1.00 | 1.00 | 0.94 | 1.00 | 0.96 | 1.00 | 1.00 | 1.00 | 0.93 | 0.99 | 1.00 | 1.00 |  |  |  |  |  |  |  |  |  |  |  |  |  |  |  |
| Mid-shelf | T2 | N2 | 1.00 | 1.00 | 0.99 | 1.00 | 0.99 | 1.00 | 1.00 | 1.00 | 0.65 | 1.00 | 1.00 | 1.00 | 1.00 |  |  |  |  |  |  |  |  |  |  |  |  |  |  |
| Mid-shelf | T2 | N3 | 1.00 | 1.00 | 0.99 | 1.00 | 0.99 | 1.00 | 1.00 | 0.99 | 0.53 | 1.00 | 1.00 | 1.00 | 1.00 | 1.00 |  |  |  |  |  |  |  |  |  |  |  |  |  |
| Mid-shelf | T3 | N1 | 0.94 | 0.31 | **<0.01** | 0.53 | **0.01** | 0.89 | 0.73 | 0.98 | 1.00 | 0.12 | 0.27 | 0.58 | 0.89 | 0.56 | 0.45 |  |  |  |  |  |  |  |  |  |  |  |  |
| Mid-shelf | T3 | N2 | 0.99 | 0.87 | 0.11 | 0.97 | 0.14 | 0.99 | 0.99 | 0.99 | 1.00 | 0.62 | 0.84 | 0.98 | 0.99 | 0.97 | 0.94 | 1.00 |  |  |  |  |  |  |  |  |  |  |  |
| Mid-shelf | T3 | N3 | 0.99 | 0.77 | 0.06 | 0.92 | 0.08 | 0.99 | 0.98 | 0.99 | 1.00 | 0.48 | 0.73 | 0.94 | 0.99 | 0.93 | 0.88 | 1.00 | 1.00 |  |  |  |  |  |  |  |  |  |  |
| Outer-shelf | T1 | N1 | 1.00 | 1.00 | 0.99 | 1.00 | 0.99 | 1.00 | 1.00 | 1.00 | 0.64 | 1.00 | 1.00 | 1.00 | 1.00 | 1.00 | 1.00 | 0.55 | 0.97 | 0.93 |  |  |  |  |  |  |  |  |  |
| Outer-shelf | T1 | N2 | 1.00 | 1.00 | 0.99 | 1.00 | 0.99 | 1.00 | 1.00 | 1.00 | 0.65 | 1.00 | 1.00 | 1.00 | 1.00 | 1.00 | 1.00 | 0.56 | 0.98 | 0.94 | 1.00 |  |  |  |  |  |  |  |  |
| Outer-shelf | T1 | N3 | 1.00 | 0.99 | 0.86 | 1.00 | 0.92 | 1.00 | 1.00 | 1.00 | 0.97 | 0.99 | 0.99 | 1.00 | 1.00 | 1.00 | 1.00 | 0.95 | 0.99 | 0.99 | 1.00 | 1.00 |  |  |  |  |  |  |  |
| Outer-shelf | T2 | N1 | 1.00 | 0.99 | 0.39 | 0.99 | 0.48 | 1.00 | 0.99 | 1.00 | 0.99 | 0.94 | 0.99 | 0.99 | 1.00 | 0.99 | 0.99 | 0.99 | 1.00 | 1.00 | 0.99 | 0.99 | 1.00 |  |  |  |  |  |  |
| Outer-shelf | T2 | N2 | 0.99 | 0.96 | 0.20 | 0.99 | 0.27 | 0.99 | 0.99 | 1.00 | 0.99 | 0.81 | 0.95 | 0.99 | 0.99 | 0.99 | 0.98 | 0.99 | 1.00 | 1.00 | 0.99 | 0.99 | 0.99 | 1.00 |  |  |  |  |  |
| Outer-shelf | T2 | N3 | 1.00 | 0.99 | 0.39 | 0.99 | 0.49 | 1.00 | 0.99 | 1.00 | 0.99 | 0.94 | 0.99 | 0.99 | 1.00 | 0.99 | 0.99 | 0.99 | 1.00 | 1.00 | 0.99 | 0.99 | 1.00 | 1.00 | 1.00 |  |  |  |  |
| Outer-shelf | T3 | N1 | 0.97 | 0.38 | **0.01** | 0.61 | **0.02** | 0.93 | 0.80 | 0.99 | 1.00 | 0.16 | 0.34 | 0.66 | 0.93 | 0.63 | 0.52 | 1.00 | 1.00 | 1.00 | 0.63 | 0.63 | 0.97 | 0.99 | 0.99 | 0.99 |  |  |  |
| Outer-shelf | T3 | N2 | 0.99 | 0.84 | 0.08 | 0.95 | 0.12 | 0.99 | 0.99 | 0.99 | 1.00 | 0.57 | 0.81 | 0.97 | 0.99 | 0.96 | 0.93 | 1.00 | 1.00 | 1.00 | 0.96 | 0.96 | 0.99 | 1.00 | 1.00 | 1.00 | 1.00 |  |  |
| Outer-shelf | T3 | N3 | 0.99 | 0.68 | **0.04** | 0.87 | 0.06 | 0.99 | 0.96 | 0.99 | 1.00 | 0.38 | 0.64 | 0.90 | 0.99 | 0.89 | 0.81 | 1.00 | 1.00 | 1.00 | 0.88 | 0.89 | 0.99 | 1.00 | 1.00 | 1.00 | 1.00 | 1.00 |  |

**Table S5**. Tukey’s HSD *post hoc* test of the pairwise comparisons of **survivorship** among populations collected from inner-, mid- and outer-shelf reefs exposed to different *temperature*nitrate* conditions under **low light** levels. The bold numbers indicated significant pairwise comparisons (P-value < 0.05). T1, T2 and T3 represents temperature treatments of 24 °C, 27 °C and 30°C, respectively. N1, N2 and N3 stand for 0.09 uM, 1.5 and 3.2 uM NO_3_^1-^, respectively.

| LOW LIGHT | | | Inner-shelf | Inner-shelf | Inner-shelf | Inner-shelf | Inner-shelf | Inner-shelf | Inner-shelf | Inner-shelf | Inner-shelf | Mid-shelf | Mid-shelf | Mid-shelf | Mid-shelf | Mid-shelf | Mid-shelf | Mid-shelf | Mid-shelf | Mid-shelf | Outer-shelf | Outer-shelf | Outer-shelf | Outer-shelf | Outer-shelf | Outer-shelf | Outer-shelf | Outer-shelf | Outer-shelf |
| --- | --- | --- | --- | --- | --- | --- | --- | --- | --- | --- | --- | --- | --- | --- | --- | --- | --- | --- | --- | --- | --- | --- | --- | --- | --- | --- | --- | --- | --- |
|  |  |  | T1 | T1 | T1 | T2 | T2 | T2 | T3 | T3 | T3 | T1 | T1 | T1 | T2 | T2 | T2 | T3 | T3 | T3 | T1 | T1 | T1 | T2 | T2 | T2 | T3 | T3 | T3 |
|  |  |  | N1 | N2 | N3 | N1 | N2 | N3 | N1 | N2 | N3 | N1 | N2 | N3 | N1 | N2 | N3 | N1 | N2 | N3 | N1 | N2 | N3 | N1 | N2 | N3 | N1 | N2 | N3 |
| Inner-shelf | T1 | N1 |  |  |  |  |  |  |  |  |  |  |  |  |  |  |  |  |  |  |  |  |  |  |  |  |  |  |  |
| Inner-shelf | T1 | N2 | 1.00 |  |  |  |  |  |  |  |  |  |  |  |  |  |  |  |  |  |  |  |  |  |  |  |  |  |  |
| Inner-shelf | T1 | N3 | 1.00 | 1.00 |  |  |  |  |  |  |  |  |  |  |  |  |  |  |  |  |  |  |  |  |  |  |  |  |  |
| Inner-shelf | T2 | N1 | 1.00 | 1.00 | 1.00 |  |  |  |  |  |  |  |  |  |  |  |  |  |  |  |  |  |  |  |  |  |  |  |  |
| Inner-shelf | T2 | N2 | 1.00 | 1.00 | 1.00 | 1.00 |  |  |  |  |  |  |  |  |  |  |  |  |  |  |  |  |  |  |  |  |  |  |  |
| Inner-shelf | T2 | N3 | 1.00 | 1.00 | 0.99 | 1.00 | 0.99 |  |  |  |  |  |  |  |  |  |  |  |  |  |  |  |  |  |  |  |  |  |  |
| Inner-shelf | T3 | N1 | 1.00 | 1.00 | **0.03** | 1.00 | **0.03** | 0.77 |  |  |  |  |  |  |  |  |  |  |  |  |  |  |  |  |  |  |  |  |  |
| Inner-shelf | T3 | N2 | 1.00 | 1.00 | 0.05 | 1.00 | 0.05 | 0.85 | 1.00 |  |  |  |  |  |  |  |  |  |  |  |  |  |  |  |  |  |  |  |  |
| Inner-shelf | T3 | N3 | 1.00 | 1.00 | **<0.01** | 1.00 | **<0.01** | **<0.01** | **<0.01** | <0.01 |  |  |  |  |  |  |  |  |  |  |  |  |  |  |  |  |  |  |  |
| Mid-shelf | T1 | N1 | 1.00 | 1.00 | 1.00 | 1.00 | 1.00 | 1.00 | 1.00 | 1.00 | 1.00 |  |  |  |  |  |  |  |  |  |  |  |  |  |  |  |  |  |  |
| Mid-shelf | T1 | N2 | 1.00 | 1.00 | 1.00 | 1.00 | 1.00 | 1.00 | 1.00 | 1.00 | 1.00 | 1.00 |  |  |  |  |  |  |  |  |  |  |  |  |  |  |  |  |  |
| Mid-shelf | T1 | N3 | 1.00 | 1.00 | **<0.01** | 1.00 | **<0.01** | 0.06 | 0.99 | 0.99 | **<0.01** | 1.00 | 1.00 |  |  |  |  |  |  |  |  |  |  |  |  |  |  |  |  |
| Mid-shelf | T2 | N1 | 1.00 | 1.00 | **<0.01** | 1.00 | **<0.01** | **<0.01** | **0.01** | **0.01** | **<0.01** | 1.00 | 1.00 | 0.51 |  |  |  |  |  |  |  |  |  |  |  |  |  |  |  |
| Mid-shelf | T2 | N2 | 1.00 | 1.00 | **<0.01** | 1.00 | **<0.01** | 0.17 | 1.00 | 1.00 | **<0.01** | 1.00 | 1.00 | 1.00 | 0.25 |  |  |  |  |  |  |  |  |  |  |  |  |  |  |
| Mid-shelf | T2 | N3 | 1.00 | 1.00 | **<0.01** | 1.00 | **<0.01** | **<0.01** | 0.89 | 0.82 | **<0.01** | 1.00 | 1.00 | 1.00 | 0.97 | 1.00 |  |  |  |  |  |  |  |  |  |  |  |  |  |
| Mid-shelf | T3 | N1 | 1.00 | 1.00 | **<0.01** | 1.00 | **<0.01** | **<0.01** | **0.03** | **0.02** | **<0.01** | 1.00 | 1.00 | 0.67 | 1.00 | 0.38 | 0.99 |  |  |  |  |  |  |  |  |  |  |  |  |
| Mid-shelf | T3 | N2 | 1.00 | 1.00 | **<0.01** | 1.00 | **<0.01** | **<0.01** | **<0.01** | **<0.01** | 0.19 | 1.00 | 1.00 | **<0.01** | **0.01** | **<0.01** | **<0.01** | **<0.01** |  |  |  |  |  |  |  |  |  |  |  |
| Mid-shelf | T3 | N3 | 1.00 | 1.00 | **<0.01** | 1.00 | **<0.01** | **<0.01** | **<0.01** | **<0.01** | 0.93 | 1.00 | 1.00 | **<0.01** | **<0.01** | **<0.01** | **<0.01** | **<0.01** | 1.00 |  |  |  |  |  |  |  |  |  |  |
| Outer-shelf | T1 | N1 | 1.00 | 1.00 | 1.00 | 1.00 | 1.00 | 1.00 | 1.00 | 1.00 | 1.00 | 1.00 | 1.00 | 1.00 | 1.00 | 1.00 | 1.00 | 1.00 | 1.00 | 1.00 |  |  |  |  |  |  |  |  |  |
| Outer-shelf | T1 | N2 | 1.00 | 1.00 | 1.00 | 1.00 | 1.00 | 1.00 | 0.16 | 0.22 | **<0.01** | 1.00 | 1.00 | **<0.01** | **<0.01** | **0.01** | **<0.01** | **<0.01** | **<0.01** | **<0.01** | 1.00 |  |  |  |  |  |  |  |  |
| Outer-shelf | T1 | N3 | 1.00 | 1.00 | 0.99 | 1.00 | 0.99 | 1.00 | 0.51 | 0.61 | **<0.01** | 1.00 | 1.00 | **0.02** | **<0.01** | 0.07 | **<0.01** | **<0.01** | **<0.01** | **<0.01** | 1.00 | 1.00 |  |  |  |  |  |  |  |
| Outer-shelf | T2 | N1 | 1.00 | 1.00 | 1.00 | 1.00 | 1.00 | 0.99 | **0.03** | 0.06 | **<0.01** | 1.00 | 1.00 | **<0.01** | **<0.01** | **<0.01** | **<0.01** | **<0.01** | **<0.01** | **<0.01** | 1.00 | 1.00 | 0.99 |  |  |  |  |  |  |
| Outer-shelf | T2 | N2 | 1.00 | 1.00 | **<0.01** | 1.00 | **<0.01** | **<0.01** | **<0.01** | **<0.01** | **<0.01** | 1.00 | 1.00 | 0.10 | 1.00 | **0.03** | 0.60 | 1.00 | 0.14 | **<0.01** | 1.00 | **<0.01** | **<0.01** | **<0.01** |  |  |  |  |  |
| Outer-shelf | T2 | N3 | 1.00 | 1.00 | **<0.01** | 1.00 | **<0.01** | **<0.01** | **<0.01** | **<0.01** | 0.15 | 1.00 | 1.00 | **<0.01** | **0.02** | **<0.01** | **<0.01** | **0.01** | 1.00 | 1.00 | 1.00 | **<0.01** | **<0.01** | **<0.01** | 0.18 |  |  |  |  |
| Outer-shelf | T3 | N1 | 1.00 | 1.00 | **<0.01** | 1.00 | **<0.01** | **<0.01** | 0.09 | 0.06 | **<0.01** | 1.00 | 1.00 | 0.88 | 1.00 | 0.63 | 0.99 | 1.00 | **<0.01** | **<0.01** | 1.00 | **<0.01** | **<0.01** | **<0.01** | 1.00 | **<0.01** |  |  |  |
| Outer-shelf | T3 | N2 | 1.00 | 1.00 | **<0.01** | 1.00 | **<0.01** | **<0.01** | **<0.01** | **<0.01** | 0.09 | 1.00 | 1.00 | **<0.01** | **0.04** | **<0.01** | **<0.01** | **<0.01** | 1.00 | 0.99 | 1.00 | **<0.01** | **<0.01** | **<0.01** | 0.28 | 1.00 | **<0.01** |  |  |
| Outer-shelf | T3 | N3 | 1.00 | 1.00 | **<0.01** | 1.00 | **<0.01** | **<0.01** | **<0.01** | **<0.01** | **<0.01** | 1.00 | 1.00 | **<0.01** | **<0.01** | **<0.01** | **<0.01** | **<0.01** | **<0.01** | 1.00 | **<0.01** | **<0.01** | **<0.01** | **<0.01** | **<0.01** | **<0.01** | **<0.01** | **<0.01** |  |

**Table S6**. Tukey’s HSD *post hoc* test of the pairwise comparisons of **bleaching frequency** among populations collected from inner-, mid- and outer-shelf reefs exposed to different *temperature*nitrate* conditions under **low light** levels. The bold numbers indicated significant pairwise comparisons (P-value < 0.05). T1, T2 and T3 represents temperature treatments of 24 °C, 27 °C and 30°C, respectively. N1, N2 and N3 stand for 0.09 uM, 1.5 and 3.2 uM NO_3_^1-^, respectively.

| LOW LIGHT | | | Inner-shelf | Inner-shelf | Inner-shelf | Inner-shelf | Inner-shelf | Inner-shelf | Inner-shelf | Inner-shelf | Inner-shelf | Mid-shelf | Mid-shelf | Mid-shelf | Mid-shelf | Mid-shelf | Mid-shelf | Mid-shelf | Mid-shelf | Mid-shelf | Outer-shelf | Outer-shelf | Outer-shelf | Outer-shelf | Outer-shelf | Outer-shelf | Outer-shelf | Outer-shelf | Outer-shelf |
| --- | --- | --- | --- | --- | --- | --- | --- | --- | --- | --- | --- | --- | --- | --- | --- | --- | --- | --- | --- | --- | --- | --- | --- | --- | --- | --- | --- | --- | --- |
|  |  |  | T1 | T1 | T1 | T2 | T2 | T2 | T3 | T3 | T3 | T1 | T1 | T1 | T2 | T2 | T2 | T3 | T3 | T3 | T1 | T1 | T1 | T2 | T2 | T2 | T3 | T3 | T3 |
|  |  |  | N1 | N2 | N3 | N1 | N2 | N3 | N1 | N2 | N3 | N1 | N2 | N3 | N1 | N2 | N3 | N1 | N2 | N3 | N1 | N2 | N3 | N1 | N2 | N3 | N1 | N2 | N3 |
| Inner-shelf | T1 | N1 |  |  |  |  |  |  |  |  |  |  |  |  |  |  |  |  |  |  |  |  |  |  |  |  |  |  |  |
| Inner-shelf | T1 | N2 | **0.01** |  |  |  |  |  |  |  |  |  |  |  |  |  |  |  |  |  |  |  |  |  |  |  |  |  |  |
| Inner-shelf | T1 | N3 | 1.00 | **0.02** |  |  |  |  |  |  |  |  |  |  |  |  |  |  |  |  |  |  |  |  |  |  |  |  |  |
| Inner-shelf | T2 | N1 | **<0.01** | 1.00 | **<0.01** |  |  |  |  |  |  |  |  |  |  |  |  |  |  |  |  |  |  |  |  |  |  |  |  |
| Inner-shelf | T2 | N2 | 0.24 | 0.99 | 0.35 | 0.96 |  |  |  |  |  |  |  |  |  |  |  |  |  |  |  |  |  |  |  |  |  |  |  |
| Inner-shelf | T2 | N3 | **<0.1** | 1.00 | **<0.01** | 1.00 | 0.93 |  |  |  |  |  |  |  |  |  |  |  |  |  |  |  |  |  |  |  |  |  |  |
| Inner-shelf | T3 | N1 | **<0.1** | 1.00 | **<0.01** | 1.00 | 0.98 | 1.00 |  |  |  |  |  |  |  |  |  |  |  |  |  |  |  |  |  |  |  |  |  |
| Inner-shelf | T3 | N2 | **<0.1** | 0.99 | **<0.01** | 1.00 | 0.39 | 1.00 | 1.00 |  |  |  |  |  |  |  |  |  |  |  |  |  |  |  |  |  |  |  |  |
| Inner-shelf | T3 | N3 | **<0.1** | 0.76 | **<0.01** | 0.99 | 0.06 | 0.99 | 0.97 | 1.00 |  |  |  |  |  |  |  |  |  |  |  |  |  |  |  |  |  |  |  |
| Mid-shelf | T1 | N1 | 1.00 | 1.00 | 1.00 | 1.00 | 1.00 | 1.00 | 1.00 | 1.00 | 1.00 |  |  |  |  |  |  |  |  |  |  |  |  |  |  |  |  |  |  |
| Mid-shelf | T1 | N2 | 0.50 | 0.98 | 0.66 | 0.76 | 1.00 | 0.67 | 0.83 | 0.14 | **0.01** | 1.00 |  |  |  |  |  |  |  |  |  |  |  |  |  |  |  |  |  |
| Mid-shelf | T1 | N3 | 0.18 | 1.00 | 0.27 | 0.98 | 1.00 | 0.97 | 0.99 | 0.50 | 0.09 | 1.00 | 1.00 |  |  |  |  |  |  |  |  |  |  |  |  |  |  |  |  |
| Mid-shelf | T2 | N1 | **<0.1** | 0.99 | **<0.01** | 1.00 | 0.57 | 1.00 | 1.00 | 1.00 | 1.00 | 1.00 | 0.24 | 0.68 |  |  |  |  |  |  |  |  |  |  |  |  |  |  |  |
| Mid-shelf | T2 | N2 | **<0.1** | 1.00 | **<0.01** | 1.00 | 0.75 | 1.00 | 1.00 | 1.00 | 0.99 | 1.00 | 0.39 | 0.84 | 1.00 |  |  |  |  |  |  |  |  |  |  |  |  |  |  |
| Mid-shelf | T2 | N3 | **<0.1** | 1.00 | **<0.01** | 1.00 | 0.82 | 1.00 | 1.00 | 1.00 | 0.99 | 1.00 | 0.48 | 0.91 | 1.00 | 1.00 |  |  |  |  |  |  |  |  |  |  |  |  |  |
| Mid-shelf | T3 | N1 | **<0.1** | 1.00 | **<0.01** | 1.00 | 0.98 | 1.00 | 1.00 | 1.00 | 0.97 | 1.00 | 0.84 | 0.99 | 1.00 | 1.00 | 1.00 |  |  |  |  |  |  |  |  |  |  |  |  |
| Mid-shelf | T3 | N2 | **<0.1** | 0.99 | **<0.01** | 1.00 | 0.47 | 1.00 | 1.00 | 1.00 | 1.00 | 1.00 | 0.19 | 0.60 | 1.00 | 1.00 | 1.00 | 1.00 |  |  |  |  |  |  |  |  |  |  |  |
| Mid-shelf | T3 | N3 | **<0.1** | 0.68 | **<0.01** | 0.97 | **0.04** | 0.99 | 0.95 | 1.00 | 1.00 | 1.00 | **<0.01** | 0.06 | 1.00 | 0.99 | 0.99 | 0.95 | 1.00 |  |  |  |  |  |  |  |  |  |  |
| Outer-shelf | T1 | N1 | 1.00 | 1.00 | 1.00 | 1.00 | 1.00 | 1.00 | 1.00 | 1.00 | 1.00 | 1.00 | 1.00 | 1.00 | 1.00 | 1.00 | 1.00 | 1.00 | 1.00 | 1.00 |  |  |  |  |  |  |  |  |  |
| Outer-shelf | T1 | N2 | 0.07 | 1.00 | 0.11 | 0.99 | 1.00 | 0.99 | 1.00 | 0.82 | 0.26 | 1.00 | 1.00 | 1.00 | 0.93 | 0.98 | 0.99 | 1.00 | 0.88 | 0.21 | 1.00 |  |  |  |  |  |  |  |  |
| Outer-shelf | T1 | N3 | **0.02** | 1.00 | **0.03** | 1.00 | 1.00 | 1.00 | 1.00 | 0.99 | 0.66 | 1.00 | 0.99 | 1.00 | 0.99 | 0.99 | 1.00 | 1.00 | 0.99 | 0.57 | 1.00 | 1.00 |  |  |  |  |  |  |  |
| Outer-shelf | T2 | N1 | **<0.01** | 1.00 | **<0.01** | 1.00 | 0.99 | 1.00 | 1.00 | 1.00 | 0.94 | 1.00 | 0.90 | 0.99 | 1.00 | 1.00 | 1.00 | 1.00 | 1.00 | 0.91 | 1.00 | 1.00 | 1.00 |  |  |  |  |  |  |
| Outer-shelf | T2 | N2 | **<0.01** | 0.83 | **<0.01** | 0.99 | 0.07 | 0.99 | 0.98 | 1.00 | 1.00 | 1.00 | **0.02** | 0.12 | 1.00 | 1.00 | 0.99 | 0.98 | 1.00 | 1.00 | 1.00 | 0.32 | 0.74 | 0.97 |  |  |  |  |  |
| Outer-shelf | T2 | N3 | **<0.01** | 0.99 | **<0.01** | 1.00 | 0.47 | 1.00 | 1.00 | 1.00 | 1.00 | 1.00 | 0.19 | 0.59 | 1.00 | 1.00 | 1.00 | 1.00 | 1.00 | 1.00 | 1.00 | 0.88 | 0.99 | 1.00 | 1.00 |  |  |  |  |
| Outer-shelf | T3 | N1 | **<0.01** | 0.68 | **<0.01** | 0.97 | **0.04** | 0.99 | 0.95 | 1.00 | 1.00 | 1.00 | **<0.01** | 0.06 | 1.00 | 0.99 | 0.99 | 0.95 | 1.00 | 1.00 | 1.00 | 0.20 | 0.57 | 0.91 | 1.00 | 1.00 |  |  |  |
| Outer-shelf | T3 | N2 | **<0.01** | 0.76 | **<0.01** | 0.98 | 0.05 | 0.99 | 0.97 | 1.00 | 1.00 | 1.00 | **0.01** | 0.08 | 1.00 | 1.00 | 0.99 | 0.97 | 1.00 | 1.00 | 1.00 | 0.26 | 0.66 | 0.94 | 1.00 | 1.00 | 1.00 |  |  |
| Outer-shelf | T3 | N3 | **<0.01** | 0.59 | **<0.01** | 0.95 | **0.02** | 0.98 | 0.92 | 1.00 | 1.00 | 1.00 | **<0.01** | **0.04** | **1.00** | 0.99 | 0.99 | 0.92 | 1.00 | 1.00 | 1.00 | 0.15 | 0.49 | 0.86 | 1.00 | 1.00 | 1.00 | 1.00 |  |

**Table S7**. Tukey’s HSD *post hoc* test of the pairwise comparisons of **chlorophyll *a*** among populations collected from inner-, mid- and outer-shelf reefs exposed to different *temperature*nitrate* conditions under **low light** levels. The bold numbers indicated significant pairwise comparisons (P-value < 0.05). T1, T2 and T3 represents temperature treatments of 24 °C, 27 °C and 30°C, respectively. N1, N2 and N3 stand for 0.09 uM, 1.5 and 3.2 uM NO_3_^1-^, respectively.

| LOW LIGHT | | | Inner-shelf | Inner-shelf | Inner-shelf | Inner-shelf | Inner-shelf | Inner-shelf | Inner-shelf | Inner-shelf | Inner-shelf | Mid-shelf | Mid-shelf | Mid-shelf | Mid-shelf | Mid-shelf | Mid-shelf | Mid-shelf | Mid-shelf | Mid-shelf | Outer-shelf | Outer-shelf | Outer-shelf | Outer-shelf | Outer-shelf | Outer-shelf | Outer-shelf | Outer-shelf | Outer-shelf |
| --- | --- | --- | --- | --- | --- | --- | --- | --- | --- | --- | --- | --- | --- | --- | --- | --- | --- | --- | --- | --- | --- | --- | --- | --- | --- | --- | --- | --- | --- |
|  |  |  | T1 | T1 | T1 | T2 | T2 | T2 | T3 | T3 | T3 | T1 | T1 | T1 | T2 | T2 | T2 | T3 | T3 | T3 | T1 | T1 | T1 | T2 | T2 | T2 | T3 | T3 | T3 |
|  |  |  | N1 | N2 | N3 | N1 | N2 | N3 | N1 | N2 | N3 | N1 | N2 | N3 | N1 | N2 | N3 | N1 | N2 | N3 | N1 | N2 | N3 | N1 | N2 | N3 | N1 | N2 | N3 |
| Inner-shelf | T1 | N1 |  |  |  |  |  |  |  |  |  |  |  |  |  |  |  |  |  |  |  |  |  |  |  |  |  |  |  |
| Inner-shelf | T1 | N2 | 0.97 |  |  |  |  |  |  |  |  |  |  |  |  |  |  |  |  |  |  |  |  |  |  |  |  |  |  |
| Inner-shelf | T1 | N3 | 0.14 | 0.99 |  |  |  |  |  |  |  |  |  |  |  |  |  |  |  |  |  |  |  |  |  |  |  |  |  |
| Inner-shelf | T2 | N1 | 0.91 | 1.00 | 0.99 |  |  |  |  |  |  |  |  |  |  |  |  |  |  |  |  |  |  |  |  |  |  |  |  |
| Inner-shelf | T2 | N2 | 0.13 | 0.99 | 1.00 | 0.99 |  |  |  |  |  |  |  |  |  |  |  |  |  |  |  |  |  |  |  |  |  |  |  |
| Inner-shelf | T2 | N3 | 0.81 | 1.00 | 0.99 | 1.00 | 0.99 |  |  |  |  |  |  |  |  |  |  |  |  |  |  |  |  |  |  |  |  |  |  |
| Inner-shelf | T3 | N1 | 0.99 | 1.00 | 0.99 | 1.00 | 0.99 | 1.00 |  |  |  |  |  |  |  |  |  |  |  |  |  |  |  |  |  |  |  |  |  |
| Inner-shelf | T3 | N2 | 0.99 | 1.00 | 0.83 | 0.99 | 0.81 | 0.99 | 1.00 |  |  |  |  |  |  |  |  |  |  |  |  |  |  |  |  |  |  |  |  |
| Inner-shelf | T3 | N3 | 1.00 | 0.98 | 0.19 | 0.94 | 0.17 | 0.86 | 0.99 | 0.99 |  |  |  |  |  |  |  |  |  |  |  |  |  |  |  |  |  |  |  |
| Mid-shelf | T1 | N1 | 0.98 | 1.00 | 0.99 | 1.00 | 0.99 | 1.00 | 1.00 | 1.00 | 0.99 |  |  |  |  |  |  |  |  |  |  |  |  |  |  |  |  |  |  |
| Mid-shelf | T1 | N2 | 0.97 | 1.00 | 0.99 | 1.00 | 0.99 | 1.00 | 1.00 | 1.00 | 0.98 | 1.00 |  |  |  |  |  |  |  |  |  |  |  |  |  |  |  |  |  |
| Mid-shelf | T1 | N3 | 0.81 | 1.00 | 1.00 | 1.00 | 0.99 | 1.00 | 1.00 | 0.99 | 0.86 | 1.00 | 1.00 |  |  |  |  |  |  |  |  |  |  |  |  |  |  |  |  |
| Mid-shelf | T2 | N1 | 0.99 | 1.00 | 0.99 | 1.00 | 0.99 | 1.00 | 1.00 | 1.00 | 0.99 | 1.00 | 1.00 | 1.00 |  |  |  |  |  |  |  |  |  |  |  |  |  |  |  |
| Mid-shelf | T2 | N2 | 0.54 | 1.00 | 1.00 | 1.00 | 1.00 | 1.00 | 0.99 | 0.99 | 0.62 | 1.00 | 1.00 | 1.00 | 0.99 |  |  |  |  |  |  |  |  |  |  |  |  |  |  |
| Mid-shelf | T2 | N3 | 0.38 | 0.99 | 1.00 | 1.00 | 1.00 | 1.00 | 0.99 | 0.97 | 0.46 | 0.99 | 0.99 | 1.00 | 0.99 | 1.00 |  |  |  |  |  |  |  |  |  |  |  |  |  |
| Mid-shelf | T3 | N1 | 1.00 | 0.93 | 0.09 | 0.82 | 0.08 | 0.65 | 0.97 | 0.99 | 1.00 | 0.94 | 0.93 | 0.70 | 0.97 | 0.41 | 0.27 |  |  |  |  |  |  |  |  |  |  |  |  |
| Mid-shelf | T3 | N2 | 1.00 | 0.99 | 0.28 | 0.97 | 0.26 | 0.93 | 0.99 | 1.00 | 1.00 | 0.99 | 0.99 | 0.93 | 0.99 | 0.75 | 0.59 | 1.00 |  |  |  |  |  |  |  |  |  |  |  |
| Mid-shelf | T3 | N3 | 1.00 | 0.82 | **0.04** | 0.66 | **0.04** | 0.49 | 0.91 | 0.99 | 1.00 | 0.85 | 0.83 | 0.52 | 0.92 | 0.25 | 0.15 | 1.00 | 1.00 |  |  |  |  |  |  |  |  |  |  |
| Outer-shelf | T1 | N1 | 0.92 | 1.00 | 0.99 | 1.00 | 0.99 | 1.00 | 1.00 | 0.99 | 0.95 | 1.00 | 1.00 | 1.00 | 1.00 | 1.00 | 1.00 | 0.85 | 0.98 | 0.69 |  |  |  |  |  |  |  |  |  |
| Outer-shelf | T1 | N2 | 0.98 | 1.00 | 0.99 | 1.00 | 0.99 | 1.00 | 1.00 | 1.00 | 0.99 | 1.00 | 1.00 | 1.00 | 1.00 | 1.00 | 0.99 | 0.95 | 0.99 | 0.86 | 1.00 |  |  |  |  |  |  |  |  |
| Outer-shelf | T1 | N3 | 0.99 | 1.00 | 0.98 | 1.00 | 0.98 | 1.00 | 1.00 | 1.00 | 0.99 | 1.00 | 1.00 | 1.00 | 1.00 | 0.99 | 0.99 | 0.99 | 0.99 | 0.97 | 1.00 | 1.00 |  |  |  |  |  |  |  |
| Outer-shelf | T2 | N1 | 1.00 | 0.99 | 0.51 | 0.99 | 0.49 | 0.99 | 0.99 | 1.00 | 1.00 | 0.99 | 0.99 | 0.99 | 0.99 | 0.92 | 0.82 | 1.00 | 1.00 | 0.99 | 0.99 | 0.99 | 0.99 |  |  |  |  |  |  |
| Outer-shelf | T2 | N2 | 1.00 | 0.99 | 0.26 | 0.97 | 0.25 | 0.92 | 0.99 | 1.00 | 1.00 | 0.99 | 0.99 | 0.92 | 0.99 | 0.73 | 0.57 | 1.00 | 1.00 | 1.00 | 0.98 | 0.99 | 0.99 | 1.00 |  |  |  |  |  |
| Outer-shelf | T2 | N3 | 1.00 | 0.99 | 0.47 | 0.99 | 0.45 | 0.98 | 0.99 | 1.00 | 1.00 | 0.99 | 0.99 | 0.98 | 0.99 | 0.90 | 0.79 | 1.00 | 1.00 | 0.99 | 0.99 | 0.99 | 0.99 | 1.00 | 1.00 |  |  |  |  |
| Outer-shelf | T3 | N1 | 1.00 | 0.95 | 0.11 | 0.87 | 0.10 | 0.74 | 0.98 | 0.99 | 1.00 | 0.96 | 0.95 | 0.75 | 0.98 | 0.47 | 0.32 | 1.00 | 1.00 | 1.00 | 0.89 | 0.97 | 0.99 | 1.00 | 1.00 | 1.00 |  |  |  |
| Outer-shelf | T3 | N2 | 1.00 | 0.99 | 0.21 | 0.95 | 0.20 | 0.88 | 0.99 | 1.00 | 1.00 | 0.99 | 0.99 | 0.89 | 0.99 | 0.66 | 0.50 | 1.00 | 1.00 | 1.00 | 0.96 | 0.99 | 0.99 | 1.00 | 1.00 | 1.00 | 1.00 |  |  |
| Outer-shelf | T3 | N3 | 1.00 | 0.87 | 0.05 | 0.72 | 0.05 | 0.56 | 0.93 | 0.99 | 1.00 | 0.89 | 0.87 | 0.58 | 0.94 | 0.31 | 0.19 | 1.00 | 1.00 | 1.00 | 0.76 | 0.90 | 0.98 | 0.99 | 1.00 | 1.00 | 1.00 | 1.00 |  |
